# Characterization, identification and expression profiling of genome-wide *R*-genes in melon and their putative roles in bacterial fruit blotch resistance

**DOI:** 10.1186/s12863-020-00885-9

**Published:** 2020-07-22

**Authors:** Md. Rafiqul Islam, Mohammad Rashed Hossain, Denison Michael Immanuel Jesse, Hee-Jeong Jung, Hoy-Taek Kim, Jong-In Park, Ill-Sup Nou

**Affiliations:** 1 Department of Horticulture, Sunchon National University, Suncheon, Jeonnam 57922 Republic of Korea; 2 Department of Biotechnology, Sher-e-Bangla Agricultural University, Dhaka, 1207 Bangladesh; 3 Department of Genetics and Plant Breeding, Bangladesh Agricultural University, Mymensingh, 2202 Bangladesh

**Keywords:** BFB, Candidate gene, Expression, Resistance, Melon, NBS-LRR, qRT-PCR

## Abstract

**Background:**

Bacterial fruit blotch (BFB), a disease caused by *Acidovorax citrulli*, results in significant economic losses in melon. The causal QTLs and genes for resistance to this disease have yet to be identified. Resistance (*R*)-genes play vital roles in resistance to plant diseases. Since the complete genome sequence of melon is available and genome-wide identification of *R*-genes has been performed for this important crop, comprehensive expression profiling may lead to the identification of putative candidate genes that function in the response to BFB.

**Results:**

We identified melon accessions that are resistant and susceptible to BFB through repeated bioassays and characterized all 70 *R*-genes in melon, including their gene structures, chromosomal locations, domain organizations, motif distributions, and syntenic relationships. Several disease resistance-related domains were identified, including NBS, TIR, LRR, CC, RLK, and DUF domains, and the genes were categorized based on the domains of their encoded proteins. In addition, we profiled the expression patterns of the genes in melon accessions with contrasting levels of BFB resistance at 12 h, 1 d, 3 d, and 6 d after inoculation with *A. citrulli*. Six *R*-genes exhibited consistent expression patterns (MELO3C023441, MELO3C016529, MELO3C022157, MELO3C022146, MELO3C025518, and MELO3C004303), with higher expression levels in the resistant vs. susceptible accession.

**Conclusion:**

We identified six putative candidate *R*-genes against BFB in melon. Upon functional validation, these genes could be targeted for manipulation via breeding and biotechnological approaches to improve BFB resistance in melon in the future.

## Background

Melon (*Cucumis melo* L.) is a highly diversified eudicot diploid (2n = 2x = 24) cucurbitaceous crop with a genome size of approximately 375 Mb [1]. Melon is economically important and ranks as the 9th most cultivated horticultural crop in terms of worldwide production [2, 3]. Its sweet, musky-flavored, fleshy fruit is rich in vitamins, minerals, and health-promoting antioxidants, including ascorbic acid, carotene, folic acid, and potassium [4–6].

Melon is vulnerable to various biotic and abiotic stresses [7, 8]. Bacterial fruit blotch (BFB) is a devastating disease of melon caused by *Acidovorax citrulli*, an aerobic, mesophilic, gram-negative, rod-shaped seed-borne bacterium belonging to the beta subdivision of the Proteobacteria [9]. BFB has been reported in many countries and poses a serious threat to melon, as well as other cucurbit crops including prickly paddy melon, citron melon, cucumber, pumpkin, squash, several types of gourds, and watermelon [10–16]. BFB causes water-soaked lesions to form on cotyledons and leaves, leading to collapse and death. The lesions on fruits are small (~ 1 cm diameter), irregular, and often sunken, progressing through the rind. The lesions then become necrotic, causing decay and cracks in the fruit. These lesions expose the plant to secondary infections and cause *A. citrulli* to colonize the pulp, eventually allowing the seed to become contaminated [17]. BFB causes 80–100% losses in production under favorable environmental situations, especially during the rainy season and in regions with highly fluctuating temperatures [18, 19]. Although BFB is of great concern to farmers and seed companies, strategies for managing this disease are limited; chemical control measures are environmentally hazardous and only partially effective, and resistant commercial cultivars have not yet been developed [13, 20–24]. Host resistance represents the most cost effective and environmentally friendly approach for managing BFB [12]. However, no QTL or *R*-gene for this disease has thus far been identified in melon. Efforts to develop BFB-resistant melon genotypes would be greatly enhanced by the identification of functional *R*-genes.

Genomic studies have provided insight into the evolution of *R*-genes, which play important roles in the plant immune system in response to various pathogens and insects [25]. Plant *R*-genes encode proteins containing domains such as Nucleotide-binding site (NBS), Leucine-rich repeat (LRR), Toll/interleukin-1 receptor (TIR), Coiled-coil (CC), and Receptor-like kinase (RLK) domains [26–32]. These domains are involved in pathogen recognition, signaling, and plant innate immunity responses [26, 27, 29, 31–35]. *R*-genes have been identified in the genomes of plant species including watermelon [36], cucumber [25], rice [37, 38], Chinese cabbage [39], maize [40], wheat [41], *Arabidopsis thaliana* [42], and apple [43].

An improved assembly and annotation of the melon (*Cucumis melo* L.) reference genome identified 70 *R-*genes in melon [1, 44, 45]. In the current study, we investigated the expression patterns of *R*-genes throughout the melon genome in response to the BFB-causing bacterium *A. citrulli* in melon accessions contrasting in BFB resistance. The aim of this study was to identify putative candidate *R*-genes that confer resistance to BFB in melon.

## Results

### Genome-wide melon *R*-genes and their chromosomal distribution

**Fig. 1:**
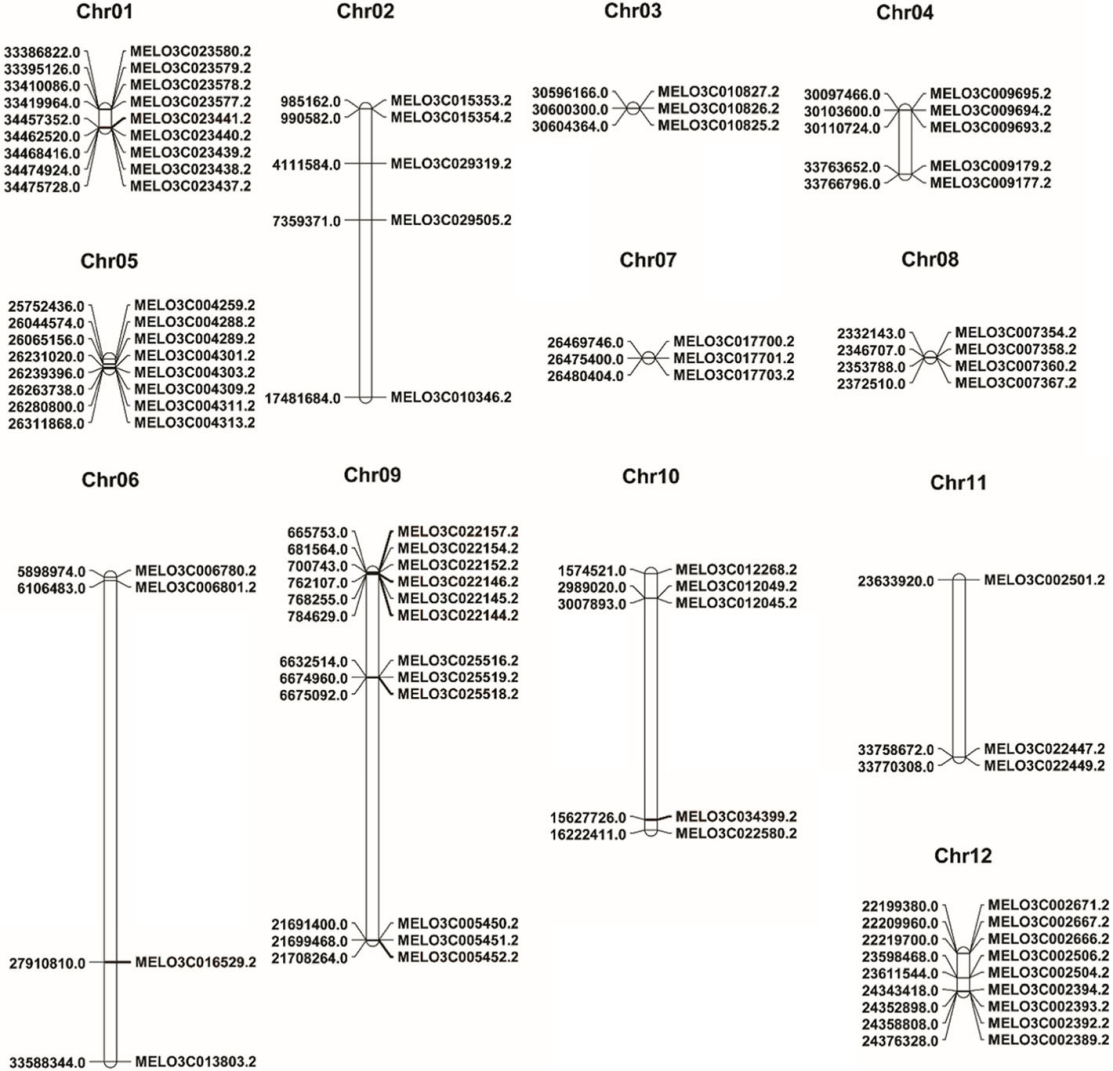
Chromosomal distribution of *R*-genes in melon genome-wide. The map was drawn using MapChart (v2.32)

The latest version (v3.6.1) of the whole-genome sequence of the melon double haploid line DHL90 was constructed using an improved assembly and annotation. This sequence contains 70 *R*-genes [1, 44]. We retrieved genomic information for these 70 *R-*genes, including their coding sequences and deduced amino acid sequences, from the cucurbit genome database (http://cucurbitgenomics.org). Detailed genomic information about these *R-*genes, including their locations on chromosomes, is provided in Table 1. Chromosomal mapping of the 70 *R*-genes revealed that they are distributed across all 12 melon chromosomes, with 1 to 12 genes per chromosome (Fig. 1; Table 1). Chromosome 9 (Chr09) contains the most *R*-genes (12), followed by Chr12 and Chr01 (10 and 9 genes, respectively). Chr11 contains the fewest *R*-genes (2), followed by Chr03 and Chr07 (3 genes each). The genes appear to be clustered, particularly in the telomere regions of chromosomes such as chr09, chr01, and chr04 (Fig. 1).

**Table 1:** Information about *R*-genes throughout the melon genome including chromosomal positions, lengths, and annotated descriptions

Sl.	Gene ID^a^	Chr. Number	Position on chromosome		CDS-length (bp)	Peptide length (AA)	Strand	Description
			Start	End				
1	MELO3C023580.2	chr01	33,386,823	33,390,698	687	288	–	Disease-resistance protein RGA2-like
2	MELO3C023579.2	chr01	33,395,126	33,397,789	2664	887	–	Disease-resistance protein RGA2-like isoform X1
3	MELO3C023578.2	chr01	33,410,087	33,414,749	1158	385	–	Disease-resistance protein
4	MELO3C023577.2	chr01	33,419,963	33,423,566	2715	904	–	Disease-resistance protein RGA2-like
5	MELO3C023441.2	chr01	34,457,351	34,462,055	2766	921	–	Receptor-kinase, putative
6	MELO3C023440.2	chr01	34,462,521	34,463,915	1338	445	–	LRR receptor-like serine/threonine-protein kinase GSO2
7	MELO3C023439.2	chr01	34,468,416	34,473,193	3207	1068	–	LRR receptor-like serine/threonine-protein kinase GSO2
8	MELO3C023438.2	chr01	34,474,924	34,475,353	336	111	+	LRR receptor-like serine/threonine-protein kinase GSO2
9	MELO3C023437.2	chr01	34,475,729	34,476,367	354	117	+	Receptor-kinase, putative
10	MELO3C029319.2	chr02	4,111,584	4,115,605	717	238	+	NBS-LRR type resistance protein
11	MELO3C015353.2	chr02	985,162	987,242	1737	578	+	Disease-resistance protein RGA2-like
12	MELO3C015354.2	chr02	990,582	993,823	3240	1080	+	Disease-resistance protein RGA2-like
13	MELO3C029505.2	chr02	7,359,371	7,363,388	765	254	–	TMV resistance protein N-like
14	MELO3C010346.2	chr02	17,481,683	17,485,283	1593	530	+	TMV resistance protein N
15	MELO3C010827.2	chr03	30,596,169	30,600,072	3663	1054	–	Receptor-kinase, putative
16	MELO3C010826.2	chr03	30,600,299	30,603,794	3054	1071	–	Receptor-kinase, putative
17	MELO3C010825.2	chr03	30,604,364	30,611,770	6069	2022	–	Receptor-kinase, putative
18	MELO3C009695.2	chr04	30,097,463	30,100,144	2682	893	+	Disease-resistance protein
19	MELO3C009694.2	chr04	30,103,601	30,106,071	2391	796	+	Disease-resistance protein
20	MELO3C009693.2	chr04	30,110,724	30,113,156	2358	786	+	Disease-resistance protein
21	MELO3C009179.2	chr04	33,763,652	33,766,776	3042	1013	+	Receptor-kinase, putative
22	MELO3C009177.2	chr04	33,766,795	33,780,875	3231	1076	+	Receptor-kinase, putative
23	MELO3C004259.2	chr05	25,752,437	25,757,292	3951	1316	+	TMV resistance protein N-like isoform X1
24	MELO3C004288.2	chr05	26,044,574	26,052,361	3171	1056	+	TMV resistance protein N-like
25	MELO3C004289.2	chr05	26,065,157	26,071,880	3867	1288	–	TMV resistance protein N-like
26	MELO3C004301.2	chr05	26,231,021	26,237,770	4032	1343	–	TMV resistance protein N-like isoform X1
27	MELO3C004303.2	chr05	26,239,395	26,244,020	2052	683	–	TMV resistance protein N-like
28	MELO3C004309.2	chr05	26,263,738	26,270,641	4134	1377	+	TMV resistance protein N-like
29	MELO3C004311.2	chr05	26,280,801	26,299,171	3156	1051	–	TMV resistance protein N-like
30	MELO3C004313.2	chr05	26,311,869	26,315,091	2115	704	–	TMV resistance protein N-like
31	MELO3C006780.2	chr06	5,898,974	5,902,420	3447	1148	–	Disease-resistance protein
32	MELO3C006801.2	chr06	6,106,483	6,109,133	846	281	–	Protein enhanced disease resistance 2-like
33	MELO3C016529.2	chr06	27,910,808	27,913,125	504	167	–	TMV resistance protein N
34	MELO3C013803.2	chr06	33,588,343	33,599,894	2184	727	+	Protein enhanced disease resistance 2
35	MELO3C017700.2	chr07	26,469,746	26,473,637	3141	1046	–	Disease-resistance protein RGA2-like
36	MELO3C017701.2	chr07	26,475,401	26,480,759	3192	1063	+	Disease-resistance protein RGA2-like
37	MELO3C017703.2	chr07	26,480,404	26,483,226	2823	940	–	Disease-resistance protein RGA2-like
38	MELO3C007354.2	chr08	2,332,143	2,335,108	1806	601	–	Cysteine-rich receptor-like protein kinase 29
39	MELO3C007358.2	chr08	2,346,707	2,353,661	4296	1431	–	Receptor-like protein kinase
40	MELO3C007360.2	chr08	23,53,788	2,361,267	4088	1395	–	Receptor-like protein kinase
41	MELO3C007367.2	chr08	2,372,510	2,386,472	4656	1551	–	Receptor-like kinase
42	MELO3C022157.2	chr09	665,753	668,864	2025	674	–	TMV resistance protein N-like isoform X1
43	MELO3C022154.2	chr09	681,564	689,908	3432	1143	–	TMV resistance protein N-like
44	MELO3C022152.2	chr09	700,743	713,705	4173	1390	+	TMV resistance protein N-like
45	MELO3C022146.2	chr09	762,107	767,613	2274	757	–	TMV resistance protein N-like
46	MELO3C022145.2	chr09	768,255	784,265	3807	1268	+	TMV resistance protein N-like
47	MELO3C022144.2	chr09	784,629	792,999	4902	1633	–	TMV resistance protein N-like
48	MELO3C025516.2	chr09	6,632,514	6,659,697	4371	1,456	–	TMV resistance protein N-like
49	MELO3C025519.2	chr09	6,674,960	6,677,738	762	253	–	Disease-resistance protein RGA2-like
50	MELO3C025518.2	chr09	6,675,092	6,676,395	648	215	–	Disease-resistance protein RGA2-like
51	MELO3C005450.2	chr09	21,691,401	21,694,271	2790	929	–	LRR receptor-like kinase family protein
52	MELO3C005451.2	chr09	21,699,468	21,702,467	3000	999	–	LRR receptor-like kinase
53	MELO3C005452.2	chr09	21,708,265	21,711,353	28,17	938	–	LRR receptor-like kinase
54	MELO3C012268.2	chr10	1,574,521	1,579,615	1800	599	+	Leaf rust 10 disease-resistance locus receptor-like protein kinase-like 1.2 isoform X4
55	MELO3C012049.2	chr10	2,989,020	2,990,934	1869	622	+	Leaf rust 10 disease-resistance locus receptor-like protein kinase-like 1.5
56	MELO3C012045.2	chr10	3,007,893	3,014,091	1503	500	–	Protein enhanced disease resistance 2
57	MELO3C034399.2	chr10	15,627,727	15,627,921	195	64	+	Disease-resistance protein At4g27190-like
58	MELO3C022580.2	chr10	16,222,411	16,222,859	447	148	–	Disease-resistance protein RGA2-like
59	MELO3C022447.2	chr11	33,758,671	33,762,610	3030	1009	–	Receptor-like protein
60	MELO3C022449.2	chr11	33,770,307	33,772,966	2145	714	–	Receptor-like protein
61	MELO3C002671.2	chr12	22,199,381	22,201,102	1350	449	+	LRR receptor-like kinase
62	MELO3C002667.2	chr12	22,209,961	22,215,123	3279	1092	+	LRR receptor-like kinase
63	MELO3C002666.2	chr12	22,219,699	22,226,478	3114	1037	+	LRR receptor-like kinase
64	MELO3C002506.2	chr12	23,598,469	23,607,646	2040	679	–	Receptor-like protein kinase
65	MELO3C002504.2	chr12	23,611,543	23,620,880	3870	1289	–	Cysteine-rich receptor-like protein kinase 28
66	MELO3C002501.2	chr12	23,633,920	23,636,908	1617	538	+	Cysteine-rich receptor-like protein kinase 26 isoform X1
67	MELO3C002394.2	chr12	24,343,418	4,346,595	2385	794	–	LRR receptor-like kinase family protein
68	MELO3C002393.2	chr12	24,352,898	4,355,087	2190	729	–	LRR receptor-like kinase
69	MELO3C002392.2	chr12	24,358,807	24,361,890	3084	1027	–	LRR receptor-like serine/threonine-protein kinase GSO1
70	MELO3C002389.2	chr12	24,376,328	24,380,811	3786	1261	+	LRR receptor-like serine/threonine-protein kinase GSO1

^a^Genomic information based on the reference Genome of Melon (DHL92) v3.6.1 retrieved from the Cucurbit Genomics Database (http://cucurbitgenomics.org)

### Gene structures, domain organizations, and motif distribution of *R-*genes in melon

We analyzed the exon–intron structures of all 70 melon *R*-genes by comparing their coding sequences with the corresponding genomic sequences using the online tool GSDS2.0 (http://gsds.cbi.pku.edu.cn/). The highest number of exons (22) was in the gene MELO3C013803, followed by 18 in MELO3C007367 (Additional file 1: Fig. S1). Among the 70 *R*-genes, 21 were mono-exonic, while 12 and 4 genes were bi- and tri-exonic, respectively.

We analyzed the conserved domains of the 70 melon *R-*genes using the Conserved Domain Database (CDD) at https://www.ncbi.nlm.nih.gov/Structure/bwrpsb/bwrpsb.cgi. We detected several disease resistance-related domains encoded by these *R*-genes, such as NB-ARC (Nucleotide-binding adaptor shared by APAF-1, R proteins, and CED-4), LRR (Leucine-rich repeat), TIR (Toll/interleukin-1 receptor), CC (Coiled-coil), and RLK (Receptor-like kinase) domains. The *R-*genes were grouped into different classes based on the presence of the following conserved domains in their encoded proteins: (i) LRR, (ii) NBS-LRR, (iii) TIR, (iv) TIR-NBS-LRR, (v) NB-ARC, (vi) CC, (vii) RLK, and (viii) DUF (Table 2 and Additional file 1: Fig. S2). Thirty-seven genes encoded proteins with only LRR domains, seven encoded proteins with NB-ARC domains, two encoded proteins with TIR domains, and only one encodes a protein with a CC domain (Table 2). Twelve genes encoded three domains (TIR, NBS, and LRR), including MELO3C004288, MELO3C004289, MELO3C004311, MELO3C004313, MELO3C022154, MELO3C022152, MELO3C022146, MELO3C022145, MELO3C022144, MELO3C004309, MELO3C004259, and MELO3C004301. A list of the genes and a description of their domains is provided in Table 2.

**Table 2:** *R*-genes throughout the melon genome categorized based on functional disease resistance-related domains

Sl.	Domain	Function	Gene ID
1	Leucine-rich repeat (LRR)	Recognition of pathogen and Plant Defense [29, 46]	MELO3C023577.2, MELO3C023579.2, MELO3C015353.2, MELO3C015354.2, MELO3C017700.2, MELO3C017701.2, MELO3C025518.2, MELO3C009695.2, MELO3C006780.2, MELO3C023441.2, MELO3C023437.2, MELO3C023440.2, MELO3C023439.2, MELO3C023438.2, MELO3C004303.2, MELO3C025516.2, MELO3C010346.2, MELO3C005450.2, MELO3C002394.2, MELO3C005451.2, MELO3C005452.2, MELO3C002671.2, MELO3C002667.2, MELO3C022447.2, MELO3C022449.2, MELO3C002392.2, MELO3C002389.2, MELO3C002393.2, MELO3C029505.2, MELO3C034399.2, MELO3C010827.2, MELO3C010826.2, MELO3C010825.2, MELO3C009179.2, MELO3C009177.2, MELO3C007367.2, MELO3C002666.2
2	Nucleotide-binding site leucine-rich repeat (NBS-LRR)	Resistance protein Signaling and Plant Defense [27, 33, 47]	MELO3C029319.2
3	Toll/interleukin-1 receptor homology (TIR)	TMV resistance protein N [34, 46]	MELO3C022157.2, MELO3C016529.2
4	Toll/interleukin-1 receptor homology nucleotide-binding site leucine-rich repeat (TIR-NBS-LRR)	Pathogen specificity and defense [34, 46, 48] {Nandety, 2013 #111}	MELO3C004288.2, MELO3C004289.2, MELO3C004311.2, MELO3C004313.2, MELO3C022154.2, MELO3C022152.2, MELO3C022146.2, MELO3C022145.2, MELO3C022144.2, MELO3C004309.2, MELO3C004259.2, MELO3C004301.2,
5	Nucleotide-binding adaptor shared by APAF-1, R proteins and CED-4 (NB-ARC)	Molecular switch in activating defenses [28, 31]	MELO3C017703.2, MELO3C025519.2, MELO3C022580.2, MELO3C023578.2, MELO3C009694.2, MELO3C009693.2, MELO3C013803.2
6	Coiled-coil domain (CC)	Pathogen recognition and signaling [31, 32, 49]	MELO3C023580.2
7	Protein kinase (RLK)	Signaling and plant defense [35, 50–52]	MELO3C007354.2, MELO3C007358.2, MELO3C007360.2, MELO3C002506.2, MELO3C012268.2, MELO3C012049.2, MELO3C002504.2, MELO3C002501.2
8	Domain of unknown function (DUF)	Protein enhanced disease resistance 2-like [53, 54]	MELO3C006801.2, MELO3C012045.2

**Fig. 2:**
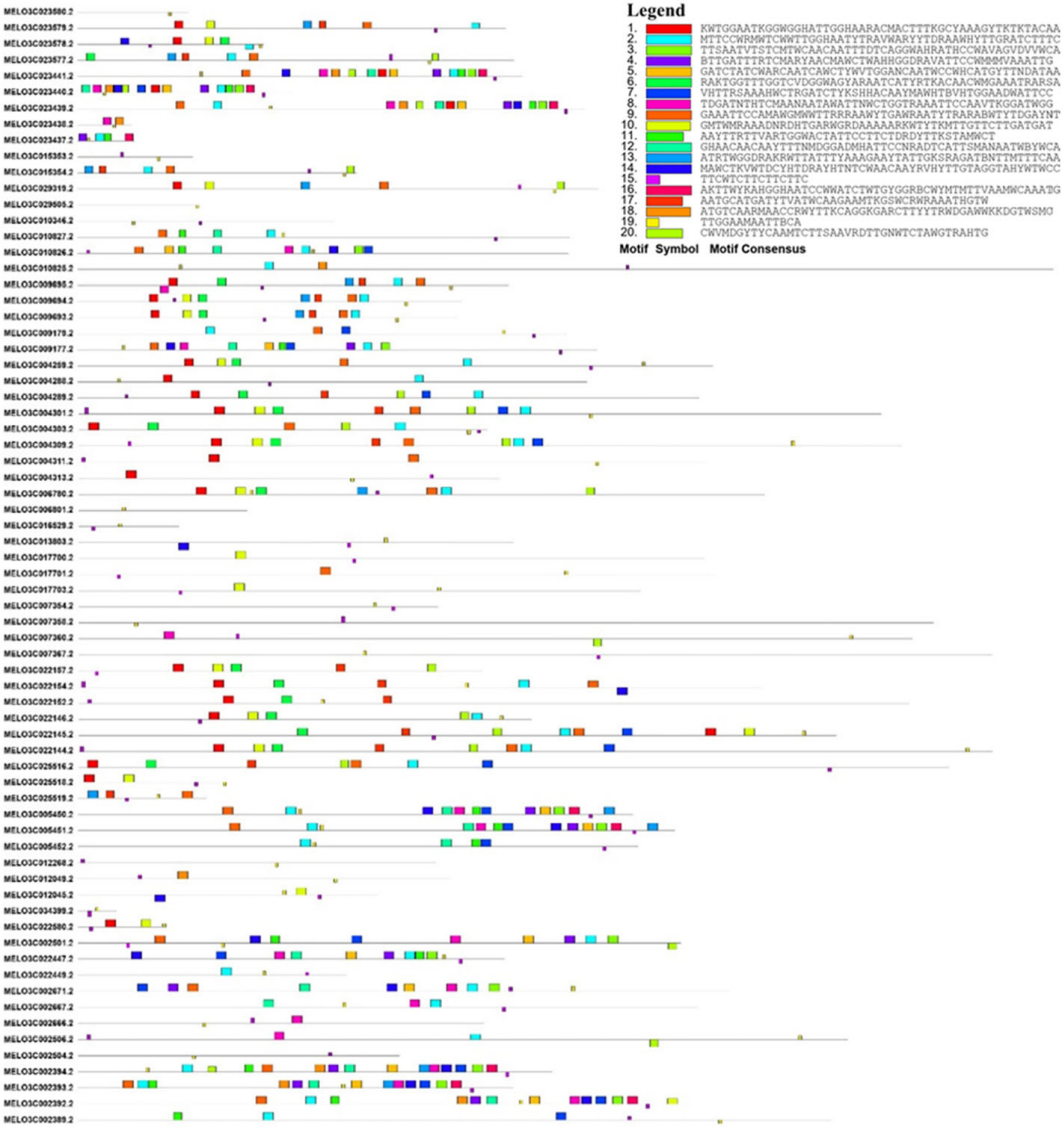
Conserved motifs in the *R*-genes of melon. Motifs are indicated by different colored rectangles. Motif sequences are provided in the legend

We analyzed the conserved motifs of these 70 *R*-genes using the MEME Suite (http://meme-uite.org/tools/meme). A total of 20 conserved motifs were detected in these 70 *R*-genes, each comprising more than 14 amino acids. The greatest number of motifs was identified in the LRR domain-encoding gene MELO3C002394, whereas the fewest were detected in MELO3C029505, MELO3C023580, and MELO3C006801, which are LRR-, CC-, and DUF-domain-encoding genes, respectively. The distribution of these conserved motifs, along with the motif sequences, is described in Fig. 2.

### Microsynteny of melon *R*-genes with genes in the watermelon and cucumber genomes

**Fig. 3:**
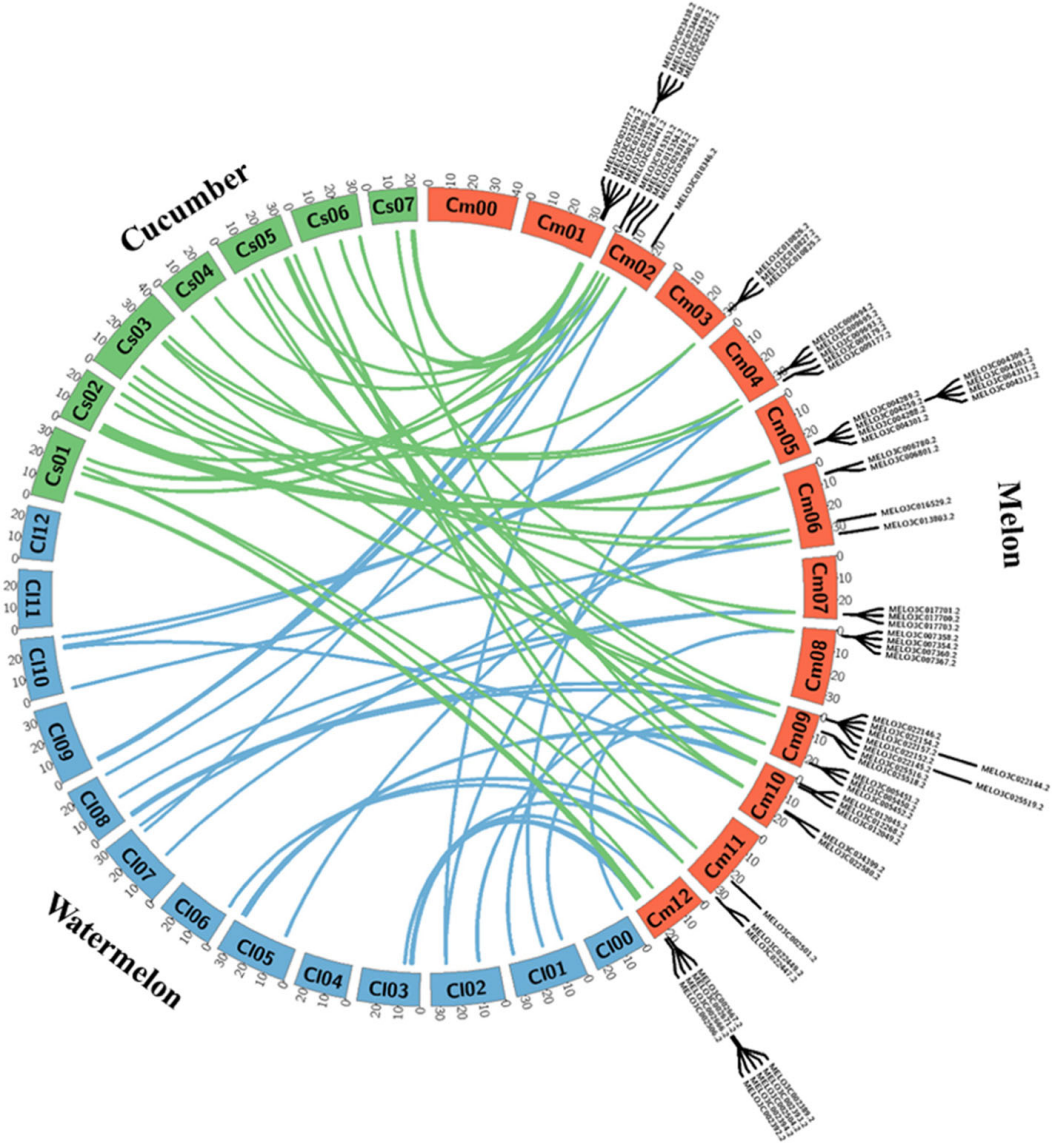
Microsynteny analysis of all 70 melon *R*-genes with those of watermelon and cucumber. Melon, watermelon, and cucumber chromosomes are shown in orange, blue, and green, respectively. The diagram was drawn using the web-based tool Circos (http://circos.ca/software/download/) circos-0.69-9.tgz

We analyzed the microsyntenic relationships of the 70 *R*-genes from melon (*Cucumis melo*) with genes in the watermelon (*Citrullus lanatus*) and cucumber (*Cucumis sativus*) genomes using the Circos tool. Most *R*-genes from melon were homologous to *R*-genes from watermelon and cucumber. However, watermelon *R*-genes on chromosomes 11 and 12 lacked homologues in melon (Fig. 3). By contrast, all 70 *R*-genes in melon had homologues in all chromosomes of cucumber.

### Expression profiles of melon *R*-genes in response to *A. citrulli* inoculation

**Fig. 4:**
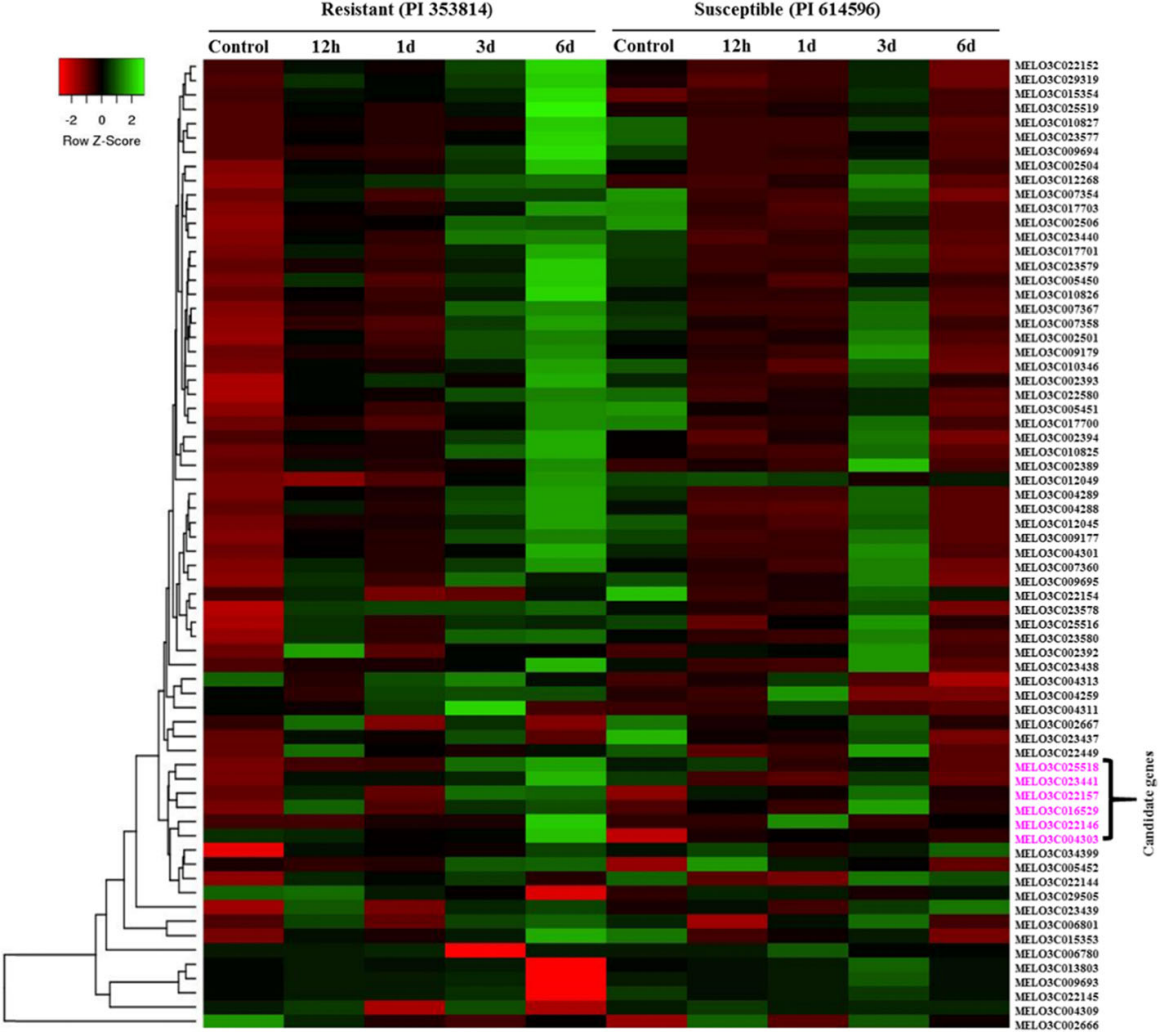
Heat map of the expression patterns of melon *R-*genes determined by qRT-PCR in BFB-resistant and -susceptible melon accessions at the indicated time points after inoculation with *A. citrulli*. The expression levels were normalized to that *Actin* (the expression levels of the *Actin* gene are shown in Additional file 1: Fig. S3). The values were obtained from the means of three biological replicates. Red and green represent the minimum and maximum values, respectively. The IDs of six putative *R*-genes are shown in pink on the right side of the figure. MELO3C002671 and MELO3C022447 were not expressed and are therefore not shown in the heatmap. The heat map was generated using the online tool Heatmapper (http://www.heatmapper.ca/expression/)

**Fig. 5:**
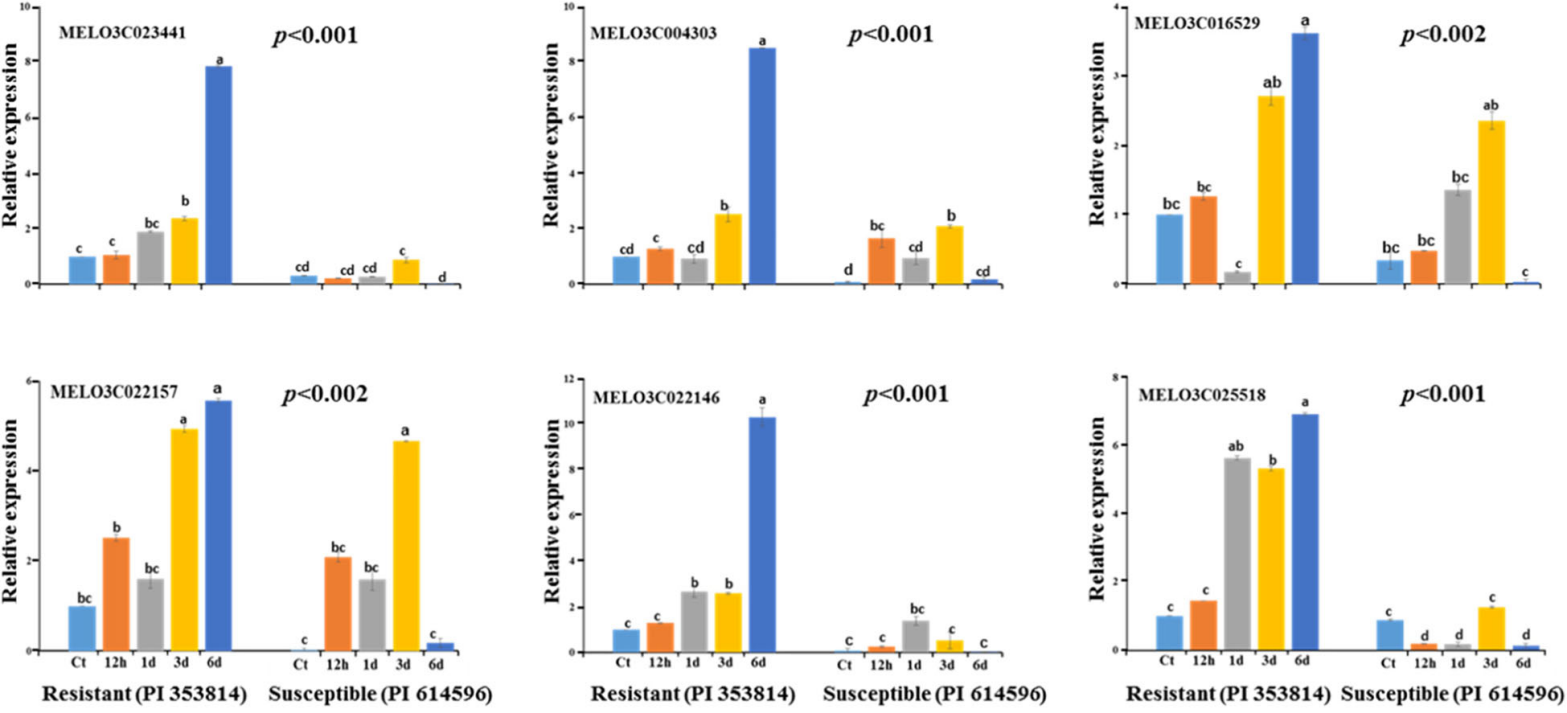
Relative expression levels of six candidate *R-*genes in resistant and susceptible melon accessions at the indicated time points after inoculation with *A. citrulli*. Error bars represent standard errors of three individual observations. Different letters above the bars indicate significant differences, as determined by Tukey’s pairwise comparison. Ct-control, h- hour, and d- day

We investigated the expression patterns of the 70 melon *R*-genes in the leaves of resistant and susceptible melon seedlings at 12 h, 1 d, 3 d, and 6 d of inoculation with *A. citrulli* strain NIHHS15–280 via qRT-PCR. Several genes showed differential expression in the resistant vs. susceptible accession at different time points. A general trend of low expression for these genes was observed in the susceptible accession (Fig. 4). On the contrary, most of the genes were significantly induced within 12 h of *A. citrulli* infection in the resistant accession and showed a general increase in expression in this accession. By contrast, in the susceptible accession, the expression of these genes fluctuated, with little or no expression at the 12 h time point. Heatmap analysis of the expression data identified a sub-cluster of six genes (MELO3C023441, MELO3C016529, MELO3C022157, MELO3C022146, MELO3C025518, and MELO3C004303) that showed contrasting trends of expression in the resistant vs. susceptible accession, with progressively increasing expression after inoculation with *A. citrulli* in the resistant but not the susceptible accession (Fig. 4). Extensive analysis of these six genes indicated that the expression of four genes (MELO3C023441, MELO3C004303, MELO3C022146, and MELO3C025518) increased in the resistant accession with increasing time after inoculation with *A. citrulli* (Fig. 5). In the susceptible accession, the expressions of these genes were very low in the initial hours after inoculation and did not show significant increase over time after inoculation. In the resistant accession, the expression of these four genes (MELO3C023441, MELO3C004303, MELO3C022146, and MELO3C025518) peaked at 6 d after inoculation, with levels approximately 8-, 8-, 10-, and 7-fold those of the control samples, respectively. In the susceptible accession, the expression of two of these genes did not increase in response to *A. citrulli* inoculation, whereas the expression of two genes (MELO3C022157 and MELO3C016529) generally increased in response to inoculation, but to a lesser extent than in the resistant accession**.** The expression of these two genes increased until 3 d after inoculation (5-fold in MELO3C016529 and 2.5-fold in MELO3C022157), followed by a decrease to their lowest levels at 6 d post inoculation (Fig. 5).

## Discussion

Here, we identified *R*-genes with putative roles in resistance to BFB disease in melon by profiling the genome-wide expression patterns of *R*-genes from melon in response to inoculation with *A. citrulli*. Disease resistance in plants involves the interaction between specific disease resistance (*R*)*-*genes in plants and avirulence (*avr*) genes of the pathogen which is known as gene-for-gene model [55, 56]. Most plant *R*-genes belong to a superfamily of genes encoding proteins with an NBS or LRR domain, an N-terminal TIR or CC domain, or an RLK/RLP domain [29, 57]. A meta-analysis of the 314 cloned plant *R*-genes revealed that 191 (61%) such genes are NBS-LRR genes and 60 (19%) genes are RLKs/RLPs [58]. NBS domains bind to and hydrolyze adenosine triphosphate (ATP) or guanosine triphosphate (GTP) and are involved in signaling; LRR domains are highly adaptable structural domains that are responsible for protein–protein interactions and play an important role in plant–pathogen recognition [59]; TIR domains provide pathogen specificity and plant defense responses, while CC domains are involved in pathogen recognition and signaling; and RLK domains play roles in signaling and plant defense responses.

In melon, four resistance gene homologue sequences were previously reported that contained 14 TIR-NBS-LRR genes [60, 61]. A study of the first complete genome sequence of melon identified 411 putative *R*-genes, including 161 RLKs, 110 RLP (receptor-like proteins) genes, 19 RLK-GNK2 (kinases containing an additional antifungal protein ginkbilobin-2 domain) genes, and 81 genes containing canonical resistance domains, such as NBS, LRR, and TIR domains [1]. Among these genes, 25 were homologous to *Pto* genes from tomato and 15 were homologous to *Mlo* genes from barley [62, 63]. After further improvements in the assembly and annotation of the melon (*Cucumis melo* L.) reference genome, 70 *R*-genes were ultimately identified in melon [44].

Our comprehensive *in-silico* analysis of the 70 melon *R-*genes revealed that they encode proteins with several disease resistance-related domains, including LRR, NBS, TIR, NB-ARC, CC, RLK, and DUF domains (Table 2). These genes are distributed across all melon chromosomes, and some are clustered in the telomeric regions of a few chromosomes (Fig. 1). The clustering of *R*-genes is an evolutionarily conserved defense mechanism in plants wherein recombination in closely located genes creates new motif combinations, which generates novel resistance specificities and broadens plant resistance to different diseases [42, 64]. *R*-gene clusters that provide resistance to multiple diseases have been reported for angular leaf spot, downy mildew, and anthracnose diseases in cucumber [65] and for blackleg, *sclerotinia* stem rot, and clubroot diseases in *B. napus* [66–68] and *B. rapa* [66]. In melon, a 1 Mb region on chromosome five contains the highest density of *R*-genes [69]. In addition, a cluster of 13 TNL genes is located in the same region as the melon *Vat* resistance gene [70], and another cluster of 7 TNL genes is located in the region harboring the *Fom-1* resistance gene [71]. The *Vat* locus encodes a CC-NBS-LRR protein that confers resistance to aphid and aphid-mediated viruses in melon. The loss of two highly conserved LRRs is linked with susceptibility to these viruses [72]. In addition, the *Fusarium* wilt resistance locus *Fom-2* is a TIR-NBS-LRR gene [73]. Expression patterns of the genome-wide *R*-genes are thus studied to identify any potential candidate genes against *A. citrulli*.

Six melon genes were highly expressed in the BFB-resistant accession. Of these genes, three (MELO3C016529, MELO3C022157, and MELO3C022146) are TNL genes, two (MELO3C023441 and MELO3C025518) are LRR genes, and one (MELO3C005452) is an NBS-LRR gene (Table 2). These genes were highly expressed at 6 d after inoculation (Fig. 5), which is consistent with our observation that BFB symptoms first appeared at 6–7 d in a susceptible accession [74].

Expression analysis upon infection with *A. citrulli* indicated a general trend of low expression for most *R*-genes in susceptible accession. By contrast, a set of genes including MELO3C023441, MELO3C004303, MELO3C022146, and MELO3C025518 were expressed at much higher levels, and MELO3C022157 and MELO3C016529 were expressed at relatively higher levels, (Fig. 5) in the resistant accession. Such higher expression in response to *A. citrulli* in the resistant accession indicates the potential involvement of these *R*-genes in BFB resistance in melon.

Several comparative transcriptomic studies have been reported in melon [75–77], but few studies have focused exclusively on expression profiling of *R*-genes against phytopathogenic agents in melon. For example, RNA-seq assessment of the changes in transcript levels at different time points in *Phytophthora capsici*-inoculated tissues of resistant and susceptible melon genotypes provided a basis for identifying candidate resistant genes [78]. Comparative transcriptome analysis identified ten genes that were differentially expressed in resistant and susceptible cultivars of melon in response to powdery mildew [79]. In addition, a study of the *MLO* (mildew resistance locus o) gene family in melon revealed candidate genes that might play roles in susceptibility to powdery mildew [80]. In watermelon, six NBS-encoding *R*-genes were identified as candidates for gummy stem blight (GSB) resistance [81, 82]. Finally, markers have been developed for detecting both GSB and BFB resistance in melon based on the sequence polymorphism in the TIR-NBS-LRR gene MELO3C022157 [81, 83]. Notably, all six candidate *R*-genes identified in the current study have corresponding homologues in watermelon and cucumber (Fig. 3). The roles of these genes in BFB resistance in these two crops remain to be investigated.

## Conclusions

We identified six putative candidate genes that might play roles in resistance to BFB in melon. This is the first report of candidate genes for BFB resistance in melon. Our findings provide a basis for further functional studies to validate the exact roles of these genes. In addition, causal sequence polymorphisms could be identified in these genes, leading to the development of markers for BFB resistance. Our findings will thus be useful for improving the BFB resistance trait in melon.

## Methods

### *A. citrulli*: collection, culture, and inoculum preparation

*A. citrulli* strain NIHHS15–280 was obtained from the National Institute of Horticultural and Herbal Science (NIHHS), South Korea. The bacterium was cultured on Petri plates containing 20 ml King’s B (KB) medium supplemented with 100 μg ml^− 1^ ampicillin for 36–48 h at 28 °C [84] until bacterial colonies formed. For all inoculations, a bacterial suspension was prepared by covering the culture plates with 5 ml of sterile, double distilled (DD) water and gently scraping the surface of the KB medium using an L-shaped rubber spreader to an optical density (OD) of 1.0 at 600 nm, as measured using a NanoDrop ND-1000 Spectrophotometer. The bacterial suspension was diluted to a final concentration of ~ 1 × 10^6^ colony forming units (cfu) mL^− 1^.

### Plant materials, growth conditions, and bioassays

The BFB-resistant (PI 353814) and -susceptible (PI 614596) melon accessions [74, 85] used in this study were obtained from the U.S. National Plant Germplasm System (https://npgsweb.ars-grin.gov/gringlobal/search.aspx), USDA, USA. The seeds were sown in a commercial nursery soil mixture in 32-cell trays and grown in a controlled plant growth chamber at 25 ± 2 °C, 16 h day length, relative humidity of 60%, and a light intensity of 440 μmoles/m^2^/s at bench level. After 3 weeks, the plants were transferred to a greenhouse.

**Fig. 6:**
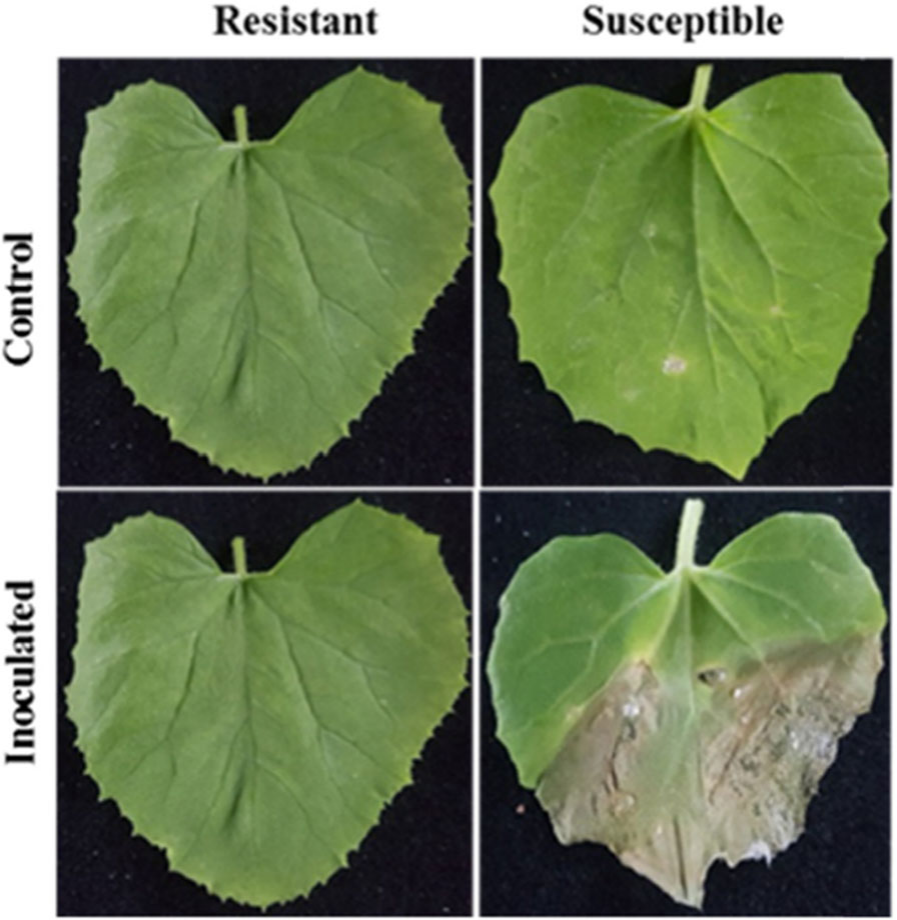
Disease symptoms in the leaves of susceptible (PI 614596) and resistant (PI 353814) melon accessions at 12 d after inoculation with *A. citrulli*. All leaves were detached from the plants immediately before they were photographed

Two weeks after germination, the plants were transferred to plastic pots and grown in a greenhouse at 24 ± 2 °C with a relative humidity of 90% where the plants were inoculated with *A. citrulli*. The resistance status of the accessions was reconfirmed via bioassay (Fig. 6) as previously reported with minor modifications [86]. Plants at the 3–5 true-leaf stage (4–5 weeks old) were sprayed with bacterial suspensions until runoff in a greenhouse at 22 ± 2 °C with a relative humidity of 96%. Plants were re-inoculated 3 d after the first inoculation to ensure that no plants had avoided inoculation and to eliminate false positives. Leaf samples from three biological replicates were collected at different time points (0 h, 12 h, 1 d, 3 d, and 6 d), immediately immersed in liquid nitrogen, and stored at − 80 °C for RNA extraction and cDNA synthesis.

### Total RNA isolation and cDNA synthesis

The melon leaves were ground to a powder in liquid nitrogen, and 100 mg of each sample with three biological replicates was subjected to total RNA extraction using the RNeasy Mini kit (Qiagen, Valencia, CA) following the manufacturer’s instructions. First-strand cDNA was synthesized from total RNA with a SuperScript III First-Strand Synthesis System kit (Invitrogen, Gaithersburg, MD).

### Identification and in silico analysis of melon *R*-genes

Genomic information for all 70 *R*-genes, as reported in the improved assembly and annotated genome of melon [44], was retrieved from the cucurbit genomic database (http://cucurbitgenomics.org) (Additional file 1: Table S1). The genes were subjected to a series of in silico analyses such as exon–intron structure, motif distribution, domain organization, chromosomal mapping, and microsynteny analyses (for specific analytical tools, see the Results section).

### Primer design and quantitative RT-PCR analysis

Gene-specific primers for quantitative RT-PCR (qRT-PCR) were designed using Primer3Plus (https://primer3plus.com/cgibin/dev/primer3plus.cgi) (Table 1). The expression patterns of the *R-*genes were analyzed by qRT-PCR in a LightCycler® instrument (Roche, Mannheim, Germany) following the manufacturer’s instructions. The reactions were performed in a 10 μL volume consisting of 5 μL of 2x qPCRBIO SyGreen Mix Lo-ROX (PCR Biosystems, London, UK), 5 pmol of primers, and cDNA template diluted to the appropriate concentrations. The PCR conditions were as follows: 5 min at 95 °C, followed by 3-step amplifications at 95 °C for 15 s, 56 °C for 15 s and 72 °C for 20 s for 45 cycles. The mean expression levels of relevant genes were calculated by the 2^–ΔΔ Ct^ method [87] using the average value of three reference genes [2, 8, 88] as internal control.

### Statistical analysis

Analysis of variance (ANOVA) and significance tests were carried out using the normalized gene expression values with MINITAB17 software (Minitab Inc., State College, PA, USA). Tukey’s pairwise comparison test was employed to determine the mean separation of expression values. *p* values indicate statistically significant variations of expression.

## Supplementary information

**Additional file 1: Table S1.** Details of the primers designed for expression profiling of melon *R*-genes. **Figure S1.** Exon–intron structures of *R*-genes in melon genome-wide. Light red rectangles and black lines indicate exons and introns, respectively. **Figure S2.** Domain structures of the 70 *R-*genes in melon. The conserved domains were identified using the NCBI Conserved Domain Database (CDD) (https://www.ncbi.nlm.nih.gov/Structure/bwrpsb/bwrpsb.cgi). Detailed descriptions of these domains are provided in Table 2. Specific domains in each protein are shown in the diagram. **Figure S3.** Gene expression profiles of resistant and susceptible melon accessions at different time points normalized to melon *Actin* expression (*CmACT7*, 149 bp), as determined by qRT-PCR analysis.

## Data Availability

We declare that the dataset(s) supporting the conclusions of this article are encompassed within the article (and its additional file(s).

## Abbreviations

*A. citrulli*: *Acidovorax citrulli*
BFB: Bacterial fruit blotch
*R*-genes: Resistance genes
*avr*: Avirulence
Fig: Figure
Chr: Chromosome
CDS: Coding Sequence
bp: Base pair
AA: Amino Acid
LRR: Leucine-rich repeat
NBS-LRR: Nucleotide-binding site leucine-rich repeat
TIR: Toll/interleukin-1 receptor homology
TIR: NBS-LRR- Toll/interleukin-1 receptor homology nucleotide-binding site leucine-rich repeat
NB-ARC: Nucleotide-binding adaptor shared by APAF-1, R proteins and CED-4
CC: Coiled-coil domain
RLK: Protein kinase
DUF: Domain of unknown function
