# Supplementary material for: Characterization, identification and expression profiling of genome-wide R-genes in melon and their putative roles in bacterial fruit blotch resistance
Source: BMC Genet. 2020 Jul 22;21:80. doi: 10.1186/s12863-020-00885-9 (PMC7376666; doi:10.1186/s12863-020-00885-9)
Supplement: Supplementary file 1 — Additional file 1: Table S1. Details of the primers designed for expression profiling of melon R-genes. Figure S1. Exon–intron structures of R-genes in melon genome-wide. Light red rectangles and black lines indicate exons and introns, respectively. Figure S2. Domain structures of the 70 R-genes in melon. The conserved domains were identified using the NCBI Conserved Domain Database (CDD) (https://www.ncbi.nlm.nih.gov/Structure/bwrpsb/bwrpsb.cgi). Detailed descriptions of these domains are provided in Table 2. Specific domains in each protein are shown in the diagram. Figure S3. Gene expression profiles of resistant and susceptible melon accessions at different time points normalized to melon Actin expression (CmACT7, 149 bp), as determined by qRT-PCR analysis. [file 12863_2020_885_MOESM1_ESM.docx]

**Supplementary materials**

**Characterization, Identification and Expression Profiling of Genome-Wide *R*-Genes in Melon and Their Putative Roles in Bacterial Fruit Blotch Resistance**

**Md. Rafiqul Islam^1,2^, Mohammad Rashed Hossain^1,3^ , Denison Michael Immanuel Jesse^1^, Hee-Jeong Jung^1^, Hoy-Taek Kim^1,*^, Jong-In Park^1^ and Ill-Sup Nou^1,*^**

**^1^**Department of Horticulture, Sunchon National University, Suncheon, Jeonnam, 57922, Republic of Korea

**^2^**Department of Biotechnology, Sher-e-Bangla Agricultural University, Dhaka 1207, Bangladesh

**^3^**Department of Genetics and Plant Breeding, Bangladesh Agricultural University, Mymensingh-2202, Bangladesh

**Authors’ email addresses:**

rafiqul@sau.edu.bd (MRI); m.r.hossain@bau.edu.bd; (MRH); michaelijesse@gmail.com (DMIJ); gml79wjd@sunchon.ac.kr (HJJ); [htkim@scnu.ac.kr](mailto:htkim@scnu.ac.kr) (HTK); jipark@sunchon.ac.kr (JIP.) and nis@scnu.ac.kr (ISN).

**^*^Correspondence:** htkim@sunchon.ac.kr (HT K); nis@sunchon.ac.kr (ISN), Tel.: +82-61-750-3249 (ISN); Fax: +82-61-750-5389 (ISN)

**Table S1.** Details of the primers designed for expression profiling of melon *R*-genes.

| **Sl.** | **Gene ID** | **Primer** | **Forward (5’-3’)** | **Reverse (5’-3’)** | **Product size (bp)** |
| --- | --- | --- | --- | --- | --- |
| 1 | MELO3C023580.2 | M3580 | CGGAGAAGCAGCAGTCCAAG | TTTGGCCAAAACCTGCCTTC | 131 |
| 2 | MELO3C023579.2 | M3579 | GAGGCTCTCCGTCGAGAAGT | GGGAAATAACATCTAGCCTC | 145 |
| 3 | MELO3C023578.2 | M3578 | GAGTGTGAGGGAGGTACGAG | CTGTCTTTCCTAATCCTCCC | 194 |
| 4 | MELO3C023577.2 | M3577 | GCAAAGTTGAAAGGTTCTCC | TCTTGTTGTAAAGTTGTGCC | 125 |
| 5 | MELO3C023441.2 | M3441 | GGGAAAGAGTTAAATGCGAC | CCATCTAGACCTTGGTTTCC | 205 |
| 6 | MELO3C023440.2 | M3440 | CCCCCTACACGACATCATGT | TGAATCTGTACCAAGTCTCC | 196 |
| 7 | MELO3C023439.2 | M3439 | GGCTCTATCACTTTACTTGG | AACTTAGATCCAACACCCTC | 118 |
| 8 | MELO3C023438.2 | M3438 | GCGGAAACATTGAGGATGCA | CGGTTTCTCGACATTTGGAC | 139 |
| 9 | MELO3C023437.2 | M3437 | CCAACTAACAGGTGATATTC | AGTGTACCAAGTTCAGGAGG | 183 |
| 10 | MELO3C029319.2 | M9319 | TGGTCCCGTAGAACGCACA | TGATGTGTGCACCCTTCGGG | 144 |
| 11 | MELO3C015353.2 | M5353 | GGGGAGAAATCAAGGAGTTC | AATTGACACAAGTCGAGTCC | 131 |
| 12 | MELO3C015354.2 | M5354 | CCGGTTTCCATGAGGAGC | CATAGGAGAACTCATCCATC | 171 |
| 13 | MELO3C029505.2 | M9505 | GGCAGGATCGAGCGAGCTTT | CTGCTCTTGCTTCAAATCGG | 177 |
| 14 | MELO3C010346.2 | M0346 | GCGCAGCAACCGTCCATTGA | CCCGCCATTCACAACAAAGC | 193 |
| 15 | MELO3C010827.2 | M0827 | CGGTACAATTCCCCCATCTT | GGTAGAGGGAACAATGCCAC | 163 |
| 16 | MELO3C010826.2 | M0826 | GGCCCCATTCCCTCCCAATT | CCCCAAAGGTTGTTGGAGGC | 146 |
| 17 | MELO3C010825.2 | M0825 | GGACCAATTCCAACCGAGCT | GAAGTGTATCACCAATATCC | 178 |
| 18 | MELO3C009695.2 | M9695 | CGTGCTCTAGTCGAATTTCT | TCTCCATACTTATGGAACCG | 141 |
| 19 | MELO3C009694.2 | M9694 | CGTGCCCCACAGTTCAAGTT | TACCAGGCTTTCCCATCCTC | 132 |
| 20 | MELO3C009693.2 | M9693 | GCGACGATTTGGATGAAATT | CATTAGTCAACGGGTAAAGC | 144 |
| 21 | MELO3C009179.2 | M9179 | GGAAGTGGGAATGTCGATGA | CCAAGGTTAATATTGTCAGC | 151 |
| 22 | MELO3C009177.2 | M9177 | CGACCCACTCAAAATCATGA | CGTAGAATCTGTTGTCTCCC | 197 |
| 23 | MELO3C004259.2 | M4259 | GGGTTCGAGGGTGTTAACAT | GCCCATGCTTTGAAGCTTCT | 151 |
| 24 | MELO3C004288.2 | M4288 | GGAATTGGAGGTATGGGCAA | AATCTCACGAAGTAGCTCCC | 156 |
| 25 | MELO3C004289.2 | M4289 | CGTGGTGTTTGGATGAATTG | AGTAGTTAAAGCATCCCTCC | 191 |
| 26 | MELO3C004301.2 | M4301 | GCGGTTTTGCCGGTGTTCTA | CAACCAGACAAACCAGCAGC | 161 |
| 27 | MELO3C004303.2 | M4303 | GGTTGGTGGACGTGATTGGT | ACTTTCCTTAAAAGCATGCC | 163 |
| 28 | MELO3C004309.2 | M4309 | GGGCCAGCTTGTTTTGCCAA | TCAGCCTCCTTCCTAGTTCC | 189 |
| 29 | MELO3C004311.2 | M4311 | GGATGGACGGTTCTTCAAAA | CATCTGCAGACATAACGTGG | 169 |
| 30 | MELO3C004313.2 | M4313 | GGTTTGGACCTGGAAGTATG | TCACAATAACGTACGGCACG | 199 |
| 31 | MELO3C006780.2 | M6780 | GGCGAGGCTAGTGCTGTTGG | GTAATCCTTTCATGAGTGGC | 179 |
| 32 | MELO3C006801.2 | M6801 | GCCGTGTCCAGAAGGTCATT | GGAAGGGGCTCTATTGATGC | 133 |
| 33 | MELO3C016529.2 | M6529 | CCGTGTGGCGGTCGGCGGTG | ACAATGCCGCCACCGTCTTC | 152 |
| 34 | MELO3C013803.2 | M3803 | CTGGGATGGATAATGGGAGG | TCAAACACTGACTCTGGAGG | 139 |
| 35 | MELO3C017700.2 | M7700 | CCCTCTTGTTCGTCATGATA | AGTTGTTTCCCTAATCTGCC | 157 |
| 36 | MELO3C017701.2 | M7701 | GCGGCGAGTATCATGGGGCT | CTCTAATACTTCAGCTTCCC | 129 |
| 37 | MELO3C017703.2 | M7703 | GTTCTACTCGATGAGCTCGC | GTACTAGCAGCAGAATAATG | 179 |
| 38 | MELO3C007354.2 | M7354 | CTCTGCCGAGGCGATCTTAT | CTACACCAAATATGGAAGAG | 154 |
| 39 | MELO3C007358.2 | M7358 | CGTACTTTTCTCCATCTCTT | TAGTAATCCTCAGCGTGGAG | 170 |
| 40 | MELO3C007360.2 | M7360 | CCAAACCAGAAAGAAGCTGC | CAGCCGGTCAAGCAAATTCC | 171 |
| 41 | MELO3C007367.2 | M7367 | GGCCGATCTAACGGTGGAAG | ACTCGGTCGACCCAAACGCC | 159 |
| 42 | MELO3C022157.2 | M2157 | GGAATCCATGGACGACGGAA | TCCCTCCACCGATGAACCTG | 156 |
| 43 | MELO3C022154.2 | M2154 | GCTAACCTCAAAGCTTGGCT | CCTCATCAGAGTTTGGGATC | 184 |
| 44 | MELO3C022152.2 | M2152 | GGAGAGAAACCGTATGGTGT | GATCCATCAACTCCTTACAC | 145 |
| 45 | MELO3C022146.2 | M2146 | GTACGGATGAACAAAAGCAT | TCCATTGTTGAACCTCCTCC | 174 |
| 46 | MELO3C022145.2 | M2145 | GGGGTATGCTACTTCCAAGT | TTCCTGACGTCTATTAATGG | 171 |
| 47 | MELO3C022144.2 | M2144 | GGCAATCACACTGGAGTTGT | AATCGGGATGTCATTCCAAC | 140 |
| 48 | MELO3C025516.2 | M5516 | GCTGATGGAGCTACGTTAAT | CTTGTTGTAACGATGACTCG | 149 |
| 49 | MELO3C025519.2 | M5519 | GCGGCATTTCTAATGGAGGG | CAACTCCTCCCATAGAAAAC | 131 |
| 50 | MELO3C025518.2 | M5518 | GGCACACGGTTTTCTTCAAC | TTCTTCTATTCTTCTGGTCC | 136 |
| 51 | MELO3C005450.2 | M5450 | GAGAGGGATGGACTTTTCCA | AAAGCCTTAGGAATTTGACC | 135 |
| 52 | MELO3C005451.2 | M5451 | CTTCCCATCGCTTGGCTTCG | ATGAAGGTGATGAGTCGACC | 132 |
| 53 | MELO3C005452.2 | M5452 | CCCAAGCTTTTTAGGTTCAA | ATCGTCATTGGTTTCCGGCC | 148 |
| 54 | MELO3C012268.2 | M2268 | GTACTCCGCCTCCGCCGGGT | ACACCACCACCGTCATCTAC | 163 |
| 55 | MELO3C012049.2 | M2049 | CGTCCCTGTTCGCCTCCTCA | CCCACTCAACTTCAAACCCC | 190 |
| 56 | MELO3C012045.2 | M2045 | GACCAGGAAATTCGGCAAGA | CAGCAGTAGAGGAGATCTCG | 174 |
| 57 | MELO3C034399.2 | M4359 | ATGGTGCAAAATATCAAGCT | CCAACTGTATACAATATCTC | 191 |
| 58 | MELO3C022580.2 | M2580 | CAGATTCCGGAGACAACCTC | GATTGAAAACCATCTTGGCC | 175 |
| 59 | MELO3C022447.2 | M2447 | CGTGTATGCGATCCAAAAGA | TCGTCGCATTTCACGCCATC | 167 |
| 60 | MELO3C022449.2 | M2449 | GCAAGGTGAAATCTCAGAGT | GAAGAACGCTTAGACTTGGG | 128 |
| 61 | MELO3C002671.2 | M2671 | CTCCCACCGACATCGCCGTCG | GTAGGAATACAAGGTGGCGA | 172 |
| 62 | MELO3C002667.2 | M2667 | CATGGGTCTCACAGGAACCT | CCACGAAGGAATCTCTCCAC | 175 |
| 63 | MELO3C002666.2 | M2666 | CCATTCCTGTATCCATTTTC | ATCTTCCAAGTTTTCCGATC | 174 |
| 64 | MELO3C002506.2 | M2506 | CAAGCTAATTTAGACCACCT | CAGTTTTTGGGGAGAAGGGT | 182 |
| 65 | MELO3C002504.2 | M2504 | GTCCCTACAAAGCCAATCTA | GAAGGGTTATTGAGTTGGTG | 177 |
| 66 | MELO3C002501.2 | M2501 | CTCGAGGTATGTATGCGTGG | CGGCGCCGTAAGAGGAGTTG | 146 |
| 67 | MELO3C002394.2 | M2394 | CTGCTCGTCTTTCTTCTTGG | CGGTTGTTCATAACCCCCAC | 161 |
| 68 | MELO3C002393.2 | M2393 | CACTCTCACCCATGTTGGGA | TGATGAATATGTGGGAGAAG | 128 |
| 69 | MELO3C002392.2 | M2392 | CCGACGAACGAGGATTCATG | CAATTACTTAAGTGCAGCTC | 169 |
| 70 | MELO3C002389.2 | M2389 | GGAGGATTGGTCTGAGAGTA | AAAGATCAAGGTGGAGCAGG | 176 |


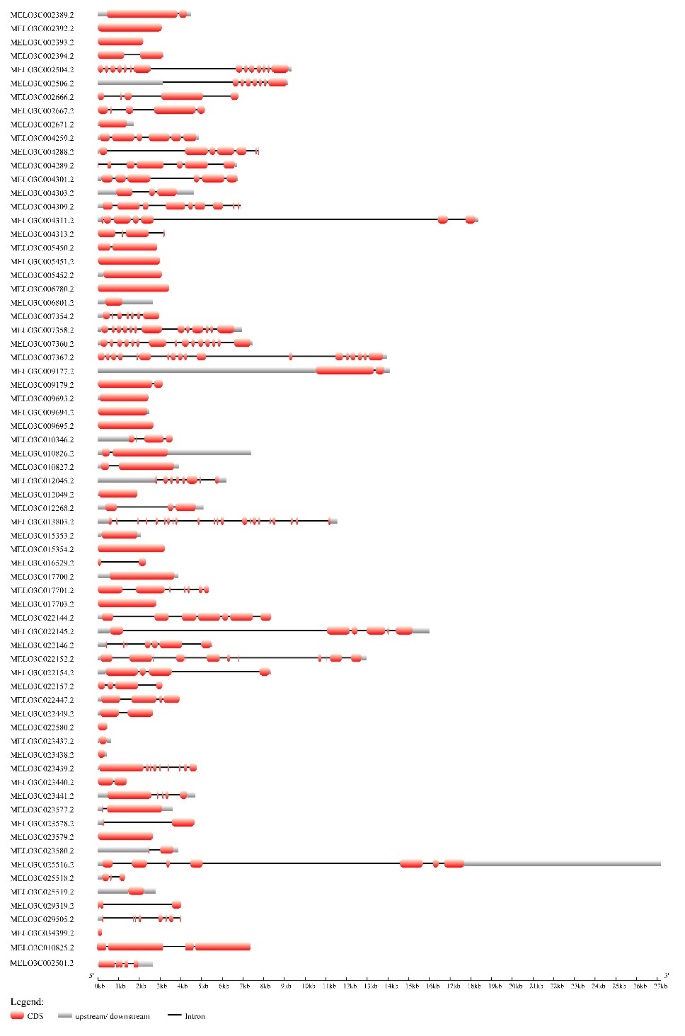


**Figure** **S1.** Exon–intron structures of *R*-genes in melon genome-wide. Light red rectangles and black lines indicate exons and introns, respectively.

**
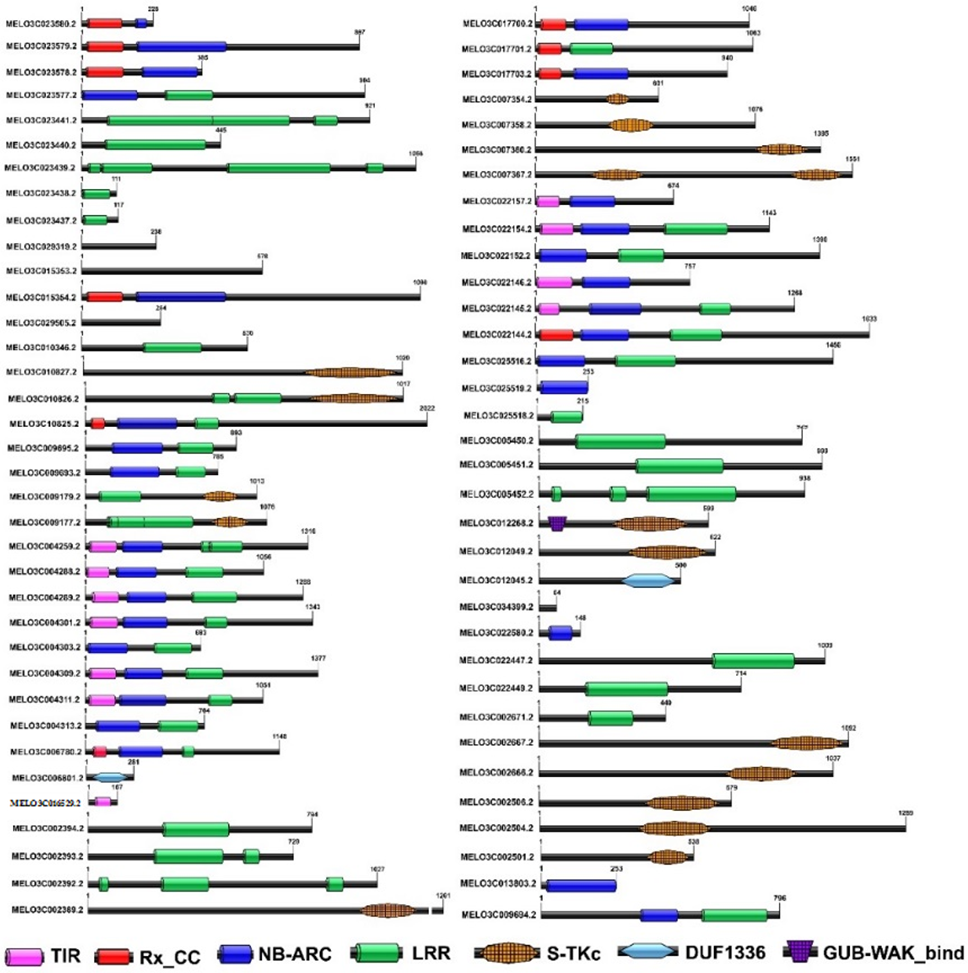
**

**Figure** **S2.** Domain structures of the 70 *R-*genes in melon. The conserved domains were identified using the NCBI Conserved Domain Database (CDD) (https://www.ncbi.nlm.nih.gov/Structure/bwrpsb/bwrpsb.cgi). Detailed descriptions of these domains are provided in Table 2. Specific domains in each protein are shown in the diagram.

**
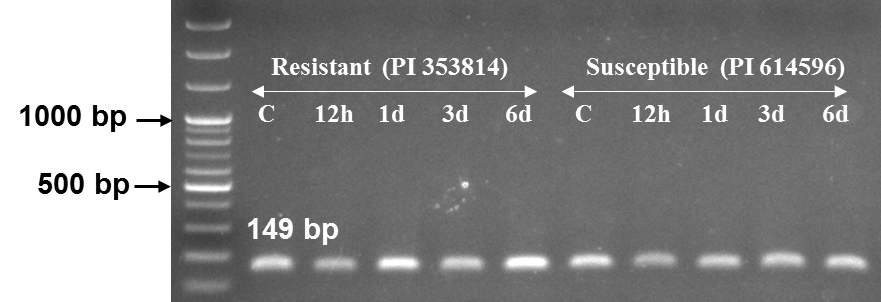
**

**Figure** **S3.** Gene expression profiles of resistant and susceptible melon accessions at different time points normalized to melon *Actin* expression (*CmACT7*, 149 bp), as determined by qRT-PCR analysis.
